# General Surgery and Pathology for Dentists

**Published:** 1895-06

**Authors:** 


GENERAL SURGERY AND PATHOLOGY FOR DENTISTS.—By Edmund W. Roughton, B. S., M. D., F. R. C. S., London, England, Warden of St. Mary's Hospital, etc., etc. With numerous original illustrations. Publishers: J. P. Segg & Co., 289-291 Regent street, London, Eng.

The author of this work of 134 pages announces that its object is to supply the student of Dentistry with an account of General Surgery and Pathology sufficiently comprehensive to enable him to practice his profession intelligently, yet concise enough to be easily mastered whilst preparing for his examination. The contents include Inflammation and its results, Bacteria in relation to disease, Wounds, Surgical fever, Shock-delirium, Hæmorrhage, Fractures, Syphilis, Tuberculosis, Tumors, Cysts, Diseases of Arteries, Diseases of Veins, Injuries and Diseases of Nerves, Diseases of Lymphatics, and Diseases of Bones. Much valuable information can be obtained from this work, the style of which is concise and comprehensive. There is every reason for believing that it will prove serviceable to the dental student, and also a means of ready reference to the dental practitioner.

